# A Comprehensive Approach to User Delegation and Anonymity within Decentralized Identifiers for IoT

**DOI:** 10.3390/s24072215

**Published:** 2024-03-29

**Authors:** Taehoon Kim, Daehee Seo, Su-Hyun Kim, Im-Yeong Lee

**Affiliations:** 1 Department of Software Convergence, Soonchunhyang University, Asan 31538, Republic of Korea; 20134101@sch.ac.kr; 2 Faculty of Artificial Intelligence and Data Engineering, Sangmyung University, Seoul 03016, Republic of Korea; daehseo@smu.ac.kr; 3 Department of Computer Software Engineering, Soonchunhyang University, Asan 31538, Republic of Korea

**Keywords:** decentralized identifier, delegation, anonymity, sequential aggregate signature, non-interactive zero-knowledge proof

## Abstract

Decentralized Identifiers have recently expanded into Internet of Things devices and are crucial in securing users’ digital identities and data. However, Decentralized Identifiers face challenges in scenarios necessitating authority delegation and anonymity, such as when dealing with legal guardianship for minors, device loss or damage, and specific medical contexts involving patient information. This paper aims to strengthen data sovereignty within the Decentralized Identifier system by implementing a secure authority delegation and anonymity scheme. It suggests optimizing verifiable presentations by utilizing a sequential aggregate signature, a Non-Interactive Zero-Knowledge Proof, and a Merkle tree to prevent against linkage and Sybil attacks while facilitating delegation. This strategy mitigates security risks related to delegation and anonymity, efficiently reduces the computational and verification efforts for signatures, and reduces the size of verifiable presentations by about 1.2 to 2 times.

## 1. Introduction

In the era rapidly evolving into a data economy, propelled by the Fourth Industrial Revolution and the ensuing interconnectedness of objects and the internet, the significance of data has become increasingly pronounced. Within this context, the vast amount of data generated by devices has necessitated advancing security and privacy measures, thus propelling the development of IdM (identity management) technologies and methodologies. Traditionally, IdM was processed within centralized systems, where a handful of central institutions or large corporations collectively managed users’ data. However, such centralized systems have become prime targets for hackers, continuously facing security threats and data breaches [1,2,3].

To address these challenges, DIDs (Decentralized Identifiers) offer a novel approach [4,5,6]. DIDs and other decentralized technologies present a new paradigm for securing personal data and protecting privacy in the data economy era [7,8,9,10,11]. It is anticipated that DIDs will transcend the limitations of traditional centralized systems by enabling user-centric data management, selective disclosure, and sovereign security. Various products, including Microsoft’s ION, Korea’s Pass, the Sovrin Foundation’s Sovrin, and ConsenSys’s uPort, have been launched, indicating widespread research and development of DIDs globally. The application of DIDs signifies a shift in personal activities, extending beyond mere individual actions to encompass various legal, economic, and social interactions, offering numerous benefits in the data economy era.

The application of DIDs in the IoT (Internet of Things) environment is particularly noteworthy. IoT devices, such as mobile devices, PCs (Personal Computers), and sensor devices, play an increasingly vital role in everyday life. These devices are key in managing and protecting users’ digital identities and personal data. The integration of DIDs in the IoT allows users to manage their identity information securely across various devices and platforms, enhancing privacy protection. This capability provides users with the autonomy to control their digital identities in the digital environment freely, enabling a secure and more personalized user experience.

Despite its advancements, DIDs encounter limitations in scenarios such as the legal guardianship of minors and the loss and damage of devices, as well as scenarios where immobile patients require guardians to obtain prescriptions on their behalf, providing patients’ EHRs (Electronic Health Records) to other medical institutions or companies, and generating power of attorney for the delegation of access rights and task execution. While traditional centralized IdM systems facilitated such delegation of authority relatively easily, DIDs must undergo further evolution to replace or integrate these functionalities to be effectively applied in various real-world service environments. Currently, DIDs struggle to support scenarios where a representative is tasked with temporarily or permanently delegating authority or identity information. This limitation underscores the need for continued development in the field of DIDs, aiming to bridge these gaps and extend the applicability of DIDs across a broader spectrum of use cases. Such improvements are crucial for ensuring that DIDs can meet the complex requirements of modern service environments, encompassing legal, healthcare, and personal data management contexts [12,13,14,15].

To address this, an approach to DIDs is exploring schemes for Holders to delegate authority to a Delegatee in a controlled manner by adding relevant information about the Delegatee to a VC (verifiable credential) and requesting its issuance from the Issuer. Furthermore, it is proposed that the delegated authority data be recorded in the VDR (Verifiable Data Registry), enabling transparent and verifiable management. However, the current scheme faces complexities in VC management when issuing new VCs or changing the authority level of a Delegatee [16,17,18].

Additionally, one of the core objectives of DIDs is to secure user privacy amidst the identifier-based relational risks that emerge from interactions across various services. The accumulation and submission of multiple VPs (verifiable presentations) bearing unique user DIDs (both Holder and Delegatee) allows attackers to link and analyze these identifiers, inferring additional personal information, thus potentially leading to privacy breaches. For instance, service providers in online shopping, social media, and healthcare sectors could correlate and track user identities, exposing details of online activities, health information, and purchase histories. Consequently, linkage attacks impinge upon the tenets of privacy and self-sovereignty [19,20,21,22,23].

Moreover, DIDs are confronted with persistent security threats within the Web 3.0 ecosystem, notably the risk of Sybil attacks. Unresolved challenges include the increasing storage demands due to the enlargement of VPs and the Delegatee’s problematic revocation of delegated authority. These challenges present critical tasks requiring resolution to uphold the system’s integrity and the users’ trust.

Therefore, this paper proposes a study on a scheme to ensure delegation and anonymity in DIDs. This paper makes the following contributions:

- Delegation and revocation by the Holder: In this paper, the Holder can delegate their authority to the delegate using a VC and an SAS (sequential aggregate signature) for delegation, and the Delegatee can prove to the Verifier that the delegation has been approved by the Holder. Additionally, this delegation uses a revocation list that allows delegates to revoke delegated authority at any time if they need it.
- Prevention of linkage and Sybil attacks: This paper generates anonymous DIDs using NIZK (Non-Interactive Zero-Knowledge) Proofs and Merkle trees. It provides user anonymity and prevents fake identities, thereby enhancing trustworthiness.
- VC management optimization and minimization of VP size: By utilizing an SAS and a reconstructed approach to VPs, this paper optimizes VC management for Holders and reduces the size of VPs by up to two times.

Section 2 explores and analyzes existing studies on the authority delegation of DIDs and the user anonymity scheme, clearly outlining the current research status and limitations. Section 3 details the various security requirements that may arise during the implementation of authority delegation and anonymity in DIDs, deriving considerations for these schemes from a security perspective. Section 4 details the proposed phases for authority delegation and anonymity, presenting their implementation and application strategies. Section 5 thoroughly analyzes and evaluates the security of the proposed scheme based on the derived security requirements. Finally, Section 6 summarizes the main contents of this paper and presents conclusions for future research directions.

## 2. Background

In this section, we delve deeply into DIDs and their significance, elaborating on the necessity and importance of authority delegation and anonymity within DIDs. Furthermore, we thoroughly analyze various schemes currently employed in the DID environment for user authority delegation and anonymity, comparing their advantages and disadvantages. Subsequently, we provide a fundamental understanding and detailed analysis of SAS and NIZK Proof schemes. Through this analysis, we clearly comprehend the current research trends and limitations of authority delegation and anonymity schemes related to DIDs, setting the stage for the necessity and distinctiveness of this proposed scheme in subsequent sections.

### 2.1. DID (Decentralized Identifier)

DIDs enable users to manage their identity information independently, without relying on central authorities or third parties, based on SSI (self-sovereign identity) principles, which emphasize personal data sovereignty.

The key characteristics of DIDs include persistence, resolvability, cryptographic verifiability, and decentralization. These features play a crucial role in ensuring the reliability and security of identity management in DIDs. In DIDs, the Holder receives a VC from the Issuer and submits it to the Verifier. The Verifier then authenticates the Holder using the verification value stored in a decentralized VDR, such as a blockchain. A critical aspect of this process is that all information exchanges occur with the user’s consent.

These DIDs are used in IoT devices such as mobile devices, PCs, and sensor devices. The application of DIDs in IoT devices is playing an increasingly important role in our daily lives, and these devices are becoming key elements in managing and protecting users’ digital identities and personal data.

**Figure 1:**
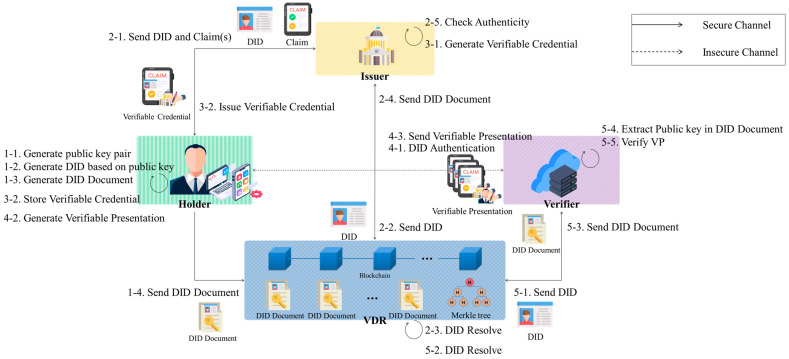
Scenario of DID.

The scenario for DIDs is illustrated in Figure 1.

- Step 1: The Holder generates a DID based on their public key and creates a DDO (DID document), incorporating their public key and DID. Subsequently, the Holder submits the DDO to the VDR for recording. Afterward, the VDR creates a Merkle tree using transactions containing the DDO and other users’ DDO transactions as input and records them on the blockchain.
- Step 2: The Holder sends its own DID and claims to the Issuer and requests a VC. Upon receiving the request, the Issuer performs DID Resolve [24] to obtain the Holder’s DDO from the VDR. The Issuer then verifies the signature in the received request message for the Holder’s VC.
- Step 3: The Issuer creates a VC comprising the Issuer and Holder’s DIDs, the Holder’s claims, and the value signed with the Issuer’s private key and then sends it to the Holder. The Holder securely stores the received VC.
- Step 4: The Holder and the Verifier perform DID Auth (DID Authentication) [25]. Upon successful verification, the Holder combines multiple VCs to create a VP and sends the generated VP to the Verifier.
- Step 5: The Verifier sends the DIDs of the Holder and Issuer to the VDR and receives the DDOs from the VDR. The Verifier then extracts the public keys and verifies the VP.

### 2.2. SAS (Sequential Aggregate Signature)

An SAS is a scheme that combines multiple digital signatures into a single signature following a specific order [26,27,28]. The primary goal of SASs is to reduce the size of signatures, yet they incorporate an additional sequential structure. This approach enables the integration of individual signatures from each user into one aggregated signature, saving storage space and transmission time. It is particularly well suited for systems where data are generated sequentially, such as blockchains, and proves beneficial in environments like auditing and logging, collaborative work, and delegation.

- $\sigma_{i}\leftarrow Sign(sk_{i},m_{i})$: Each user $u_{i}$ generates an individual signature $\sigma_{i}$ for the message $m_{i}$ using their private key $sk_{i}$.
- $\sigma_{i+1}\leftarrow AggSign(\sigma_{i},sk_{j})$: User $u_{j}$, upon receiving the previously signed $\sigma_{i}$, uses this signature and their own private key $sk_{j}$ to create the sequential aggregate signature $\sigma_{i+1}$.
- $1/0\leftarrow Verify(\sigma_{i+1},PK)$: The Verifier performs verification using the final sequentially aggregated signature $\sigma_{i+1}$ and the public key set $PK={\left\{pk_{i},\ldots ,pk_{j}\right\}}_{i,j\in \left[1,n\right]}$. If the verification is correct, it returns 1; otherwise, it returns 0.

### 2.3. NIZK (Non-Interactive Zero-Knowledge) Proof

An NIZK Proof is a cryptographic scheme that enables the owner of information to prove the possession of specific information without revealing the actual information. Unlike traditional Zero-Knowledge Proofs, an NIZK Proof does not require interaction between the prover and the verifier. Instead, the prover can demonstrate possession of the information with a single message transmission.

- $crs\leftarrow Setup(1^{\lambda })$: The prover generates a common reference string crs needed for the cryptographic protocol, given a security parameter. The crs serves as public information accessible to all participants of the protocol, containing parameters required for proof and verification.
- π←Prove(crs, x, w): The prover generates a proof value π, indicating their knowledge of a specific problem. The inputs for this proof are the proposition x to be proved, a related secret witness w known only to the prover, and crs.
- 1/0←Verify(crs,x,π): Upon receiving x and π from the prover, the verifier performs verification using these along with crs. If the verification is correct, it returns 1; otherwise, it returns 0.

### 2.4. Related Work

#### 2.4.1. Scheme of Delegation in DID

**Figure 2:**
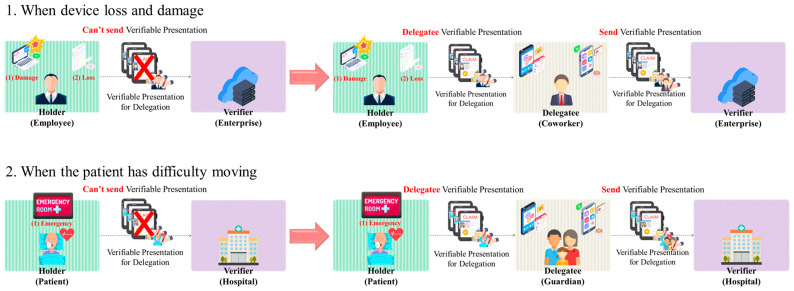
Examples and solutions of situations requiring DID-based delegation for IoT systems.

In digital identity verification, DIDs leverage the IoT to ensure user authentication and self-sovereign identity. This integration, while innovative, has its challenges. As depicted in Figure 2, certain issues arise in the operational environment of DIDs when interfaced with IoT devices. To this end, schemes that can provide delegation in DIDs are being studied [16,17,18].

Chadwick et al. proposed FIDO (Fast Identity Online) and DIDs as schemes to overcome issues in traditional ID management systems, proposing a concept model, architecture, and protocols extending FIDO’s UAF (Universal Authentication Framework), thereby providing robust authentication and powerful authorization [29]. They also developed FIDO and DIDs for UK NHS (United Kingdom National Health Service) patients, providing authority delegation and revocation. This scheme ensures privacy in authority delegation for the Holder and Delegatee by separating the public key pairs between Issuers and Verifiers.

Abdelgalil et al. proposed a framework named Health-Block for jointly sharing EHRs and personal privacy [30]. Utilizing Hyperledger Indy and IPFS (InterPlanetary File System), this framework stores and shares patient EHRs in a distributed off-chain repository, ensuring record immutability and granting patients complete control over their EHRs. It involves the Holder hashing the proof of delegating authority to the Delegatee and recording it as a transaction on the blockchain. Recording the DID as a certificate linking a series of attributes and public keys provides proof of authority delegation for the Delegatee. Additionally, Hyperledger Fabric stores access control policies (roles and actions) and delegations for patient EHRs. Roles are divided into patients, EHR providers, and EHR consumers, and actions are classified as creation, encryption, upload, access, delegate permission, and modify. However, the inclusion of Holder and Delegatee information in the VP exposes their privacy.

Dursun et al. proposed a new on-chain governance model utilizing both policy-based management and DIDs [31]. The design and implementation focused on creating a simple, fair, robust, scalable, user-friendly, and flexible framework. However, the inclusion of Holder and Delegatee information in the VC compromises their privacy.

These three schemes commonly involve the Holder sending their claims and specific Delegatee information to the Issuer when intending to delegate authority. The Holder then receives an appropriate VC from the Issuer, signs it with their private key to create a VP, and transmits the VP to the Delegatee for authority delegation. However, this process requires reissuing a VC from the Issuer when delegating to another Delegatee or changing the authority level of an existing Delegatee, causing communication and computation overhead for the Holder and Issuer. Furthermore, this approach results in complexities in VC management and overhead in VP storage capacity for the Holder. Additionally, using a unique DID singularly exposes them to linkage attacks and privacy breaches.

Except for Dursun et al.’s scheme, the other two do not provide time-stamped authority delegation or revocation within a specified period, posing a problem for Holders who wish to revoke the authority delegation to a Delegatee at a desired time. This paper analyzes these schemes and based on them, reconstructs the SAS and VPs in DIDs to provide minimal authority delegation and disconnects the DID-based linkages using NIZK Proofs to ensure users’ anonymity. Additionally, it aims to optimize VC management and minimize overhead in VP storage capacity, providing the revocation of a delegated authority.

#### 2.4.2. Scheme of Anonymity in DID

Kim’s scheme applies a blockchain-based DID to vehicles, recording vehicle modifications and maintenance history and considering security in credential authentication systems like a car birth certificate. Using a unique DID singularly could expose vehicle identifiers, driver information, driving data, and location and compromise privacy. To protect these securely, they proposed a vehicular DID ensuring anonymity using 16 bytes of multiple X.509 certificate public keys in WAVE (Wireless Access in Vehicular Environment) as an sDID (short-term DID) [32]. However, using 16 bytes for a DID is not secure from collision possibilities. Until replaced with another sDID after a certain period, the used DID remains the same, and is not completely secure from linkage attacks. Also, recording a DDO in the VDR for each sDID is inefficient.

Song’s scheme highlights that while DIDs strive to grant users complete sovereignty over digital assets within the Web3 ecosystem, this risks privacy breaches concerning user credentials and identity information, posing problems with the longstanding nemesis of Sybil attacks in Web 3.0 [21]. To solve this, they use Commitment and NIZK to prove legitimate users while hiding the DID and protecting against Sybil attacks using the Merkle tree.

Bosk et al. identifies the problem that a traditional DID exposes the Issuer’s identity to all participants, leading to data breaches. They propose a new anonymous credential using NIZK, a modified PS (Pointcheval Sanders) Signature, and Aggregate, allowing the Holder to hide the Issuer of credentials while ensuring trust in a trustless setting [33]. However, it is not secure when it comes to Sybil attacks, as attackers can create multiple fake identities and exert undue influence within the DID.

This paper analyzes these schemes and based on them, uses NIZK in a DID for authority delegation to hide the DID of the Holder and Delegatee, ensuring users’ anonymity by disconnecting DID-based linkages. Additionally, it prevents against Sybil attacks using the Merkle tree.

## 3. Security Requirements

This section details the essential security requirements and additional requirements for a comprehensive approach to user delegation and anonymity within a DID for the IoT. These requirements play a crucial role in guaranteeing the security of user delegation and anonymity in DIDs.

### 3.1. Primary Security Requirements

#### 3.1.1. Delegation

- **Authentication of Delegation**

With a DID, a Holder must be able to delegate their authority temporarily or permanently to another entity (Delegatee). To ensure the legitimacy of such authority delegation, the Delegatee must clearly prove that they have indeed been delegated the authority by the Holder. However, if the Verifier fails to verify or incorrectly verifies the Delegatee’s authority delegation, the attacker risks misusing or abusing the Holder’s authority. This can lead to personal information leakage and improper data access and manipulation. Therefore, it is essential for the Delegatee to prove their identity and clearly confirm the Holder’s intention of authority delegation.

- **Revocation of Delegatee**

The Holder should be able to revoke the delegated authority of the Delegatee at any time if necessary. In such cases, a function to verify and process legitimate requests for revocation of authority delegation is needed. If revocation measures are not properly implemented, a Delegatee whose authority has been revoked could misuse or abuse such authority. To prevent this, when an unauthorized Delegatee attempts access using their credentials, the Verifier should cryptographically verify these credentials to block unauthorized access. Additionally, it is vital to prevent illegitimate user authentication requests and ensure secure authority management.

#### 3.1.2. Anonymity

- **Prevention of Linkage Attack (Unlinkability)**

Linkage attacks are considered a significant threat to user privacy. With DIDs, although users can generate unique identifiers for each interaction, these identifiers can be traced and inferred, connecting multiple identifiers across various services to track a user’s online activities and identity. Thus, a third party collecting and analyzing multiple VPs should not be able to trace the unique DIDs of the Holder and Delegatee or infer additional personal information.

- **Prevention of Sybil Attack**

Sybil attacks are a serious problem in decentralized networks, where an individual or group can fill multiple nodes with fake or duplicated identities, disrupting or manipulating the network’s operation. Attackers can create multiple fake identities and exert abnormal influence within the network, using these identities to manipulate network decisions, voting, and resource allocation. With DIDs, it must be impossible for an individual or group to create fake or duplicated identities and disrupt or manipulate the network.

### 3.2. Additional Requirements

- **Optimization of VC Management**

For a Holder to delegate authority to a Delegatee, the VC or VP must include information proving the delegation from the Holder and about the Delegatee. Additionally, the Delegatee should submit the delegated VC or VP to the Verifier for verification. Therefore, it is crucial for the Holder and Delegatee to issue new VCs for authority delegation and manage and store the delegated VCs. However, due to varying authority levels for each Delegatee, the Holder faces inconvenience in issuing new VCs for each Delegatee. Also, Delegatees find it challenging to store and manage VCs when delegated authority from multiple Holders. Thus, the issuance of new VCs for authority delegation and the management of the delegated VCs or VPs must be optimized.

- **Minimization of VP Storage Capacity**

With DIDs, the basic structure of a VP comprises selected VCs issued by the Issuer and the Holder’s signature as input. Additionally, for delegating authority to a Delegatee, the Delegatee’s DID and signature must be included. However, if a Delegatee receives authority delegation from multiple Holders or multiple authority levels, the size and number of VCs or VPs increase, along with the burden of storage capacity. Thus, this needs to be minimized.

- **Minimization of DDO Records in VDR**

When a Holder creates a DID, it generates a DDO by binding it with the corresponding public key. The created DDO is then recorded in the VDR for future verification. However, if a new DID and DDO are created to ensure the anonymity of the Holder or Delegatee, the number of DDOs to be recorded in the VDR increases, leading to an increased burden on the VDR. Therefore, this needs to be minimized.

## 4. DID-Based Delegated Scheme with User Anonymity in IoT

**Figure 3:**
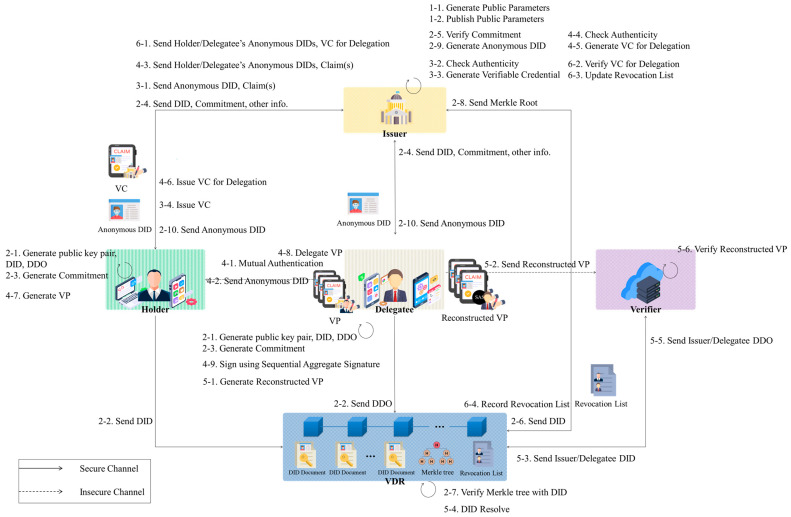
Scenario of DID-based delegated scheme with user anonymity in IoT.

This section proposes a comprehensive approach to user delegation and anonymity within DIDs for the IoT. The existing DID operational process utilizes an SAS to facilitate user authority delegation and verification, with the Delegatee receiving authentication services from the Verifier on behalf of the Holder. Additionally, NIZK and Merkle trees are employed to hide the identities of the Holder and Delegatee while enabling verification by the Verifier. This paper is structured into six phases, as illustrated in Figure 3: the setup phase, the registration phase, the VC issuance phase, the authority delegation phase, the VP verification phase, and the revocation phase.

### 4.1. System Entities

The roles of system entities are as follows:

- Holder (Delegator): The Holder, as a trustworthy entity of data sovereignty, receives verifiable credentials from the Issuer, providing authentication services to the Verifier through these credentials. The Holder is also an entity capable of temporarily or permanently delegating their authority to a Delegatee.
- Issuer: The Issuer, a government agency that acts as a trusted certification center, verifies the Holder’s credentials when a Holder requests VC issuance. If the Holder’s credentials are correct, the Issuer creates a digital signature that guarantees the Holder’s credentials can be trusted when the Holder later authenticates with a Verifier. The Issuer then issues a VC to the Holder, and the Issuer’s digital signature is attached.
- Verifier: As a service provider, the Verifier receives the Holder’s VP from the Holder when requesting authentication and verifies it. To determine if the authentication is valid, the Verifier verifies the Holder’s digital signature included in the VP to see if the owner of the VP is the Holder, and the Verifier verifies the issuer’s digital signature to see if a trusted issuer issued the VC included in the VP. If the authentication is correct, the Verifier provides the service to the Holder.
- Delegatee: The Delegatee, an entity that receives delegation from the Holder, requests authority delegation from the Holder and, upon approval, assumes the delegated authority, receiving authentication services from the Verifier on behalf of the Holder.
- VDR (Verifiable Data Registry): A VDR is a verifiable data repository that stores and manages credentials of all entities and related data, as well as a revocation list, for authentication purposes.

### 4.2. System Parameters

The system parameters used in this paper are as presented in Table 1, and they are denoted as follows.

### 4.3. Assumption

The assumptions for the comprehensive approach to user delegation and anonymity within DIDs for the IoT are as follows:

1. As a trusted entity, the Issuer generates and manages public parameters, and all channels communicating with the Issuer are assumed to be secure [34].
2. The VDR, capable of blockchain computations, stores publicly available credentials and can issue DID certificates. Additionally, the VDR maintains and publicly discloses a revocation list, accessible for verification by anyone.
3. The structure of transactions stored in the VDR is composed of {sender address, data, recipient address, sender’s signature, and sender’s public key}.
4. For delegation, the Holder utilizes a TLS (Transport Layer Security) handshake [35] for mutual authentication with the Delegatee.
5. The structure of the revocation list stored in the VDR comprises {Holder’s anonymous DID, revoked anonymous Delegatee’s DID, VC to be revoked, and revoked date}.
6. For secure cryptography in this paper, detailed cryptographic functions, hash functions, and signatures are based on large primes p,q, an arbitrary elliptic curve reference point P using the ECDSA (Elliptic Curve Digital Signature Algorithm) [36], and a hash function H(), all of which are publicly disclosed to all entities.
7. The Pedersen Commitment [37] and NIZK are used to create anonymous DIDs to provide user anonymity in this paper.
8. An additive group $G_{1}$ and a multiplicative group $G_{2}$, both with the same prime q, are generated; $g_{1}$ is defined as the generator of $G_{1}$, and $g_{2}$ as the generator of $G_{2}$. Moreover, it satisfies $e:\;G_{1}\;\times \;G_{2}\;⟶\;G_{T}$.

### 4.4. Proposed Scheme

The proposed scheme is divided into six phases, which is detailed as follows.

#### 4.4.1. Setup

In the setup phase, the Issuer generates and publishes public parameters for all DID participants (Holder, Issuer, and Delegatee). An initial empty revocation list is also created and recorded in the VDR.

- Step 1: The issuer generates the following public parameters using security parameter λ:
  (1) $$PP=\{p,\;q,\;g_{1},g_{2},G_{1},G_{2},G_{T},\;e,H,\;P,\;crs\}$$
- Step 2: The issuer initializes and creates the revocation list HL.

#### 4.4.2. Registration

In the registration phase, DID participants create their public key pairs and DIDs, requesting their recording in the VDR. The VDR then registers and publishes these participants. Anonymous DIDs are also created to ensure the anonymity of Holders and Delegatees.

- Step 1: The participants (Holder, Issuer, and Delegatee) select a prime $x_{*}$ in $Z_{p}$, use it as their private key $sk_{*}$, generate their public key $pk_{*}$ as follows, and publish $pk_{*}$ to all participants, including the VDR.
  (2) $$sk_{*}=x_{*},\;pk_{*}={sk}_{*}\cdot P$$
- Step 2: The participants create $DID_{*}$ using their generated public key $pk_{*}$ and a timestamp ts, then map $pk_{*}$, $DID_{*}$ to create a DID document $DDO_{*}$ and sign it based on the ECDSA.
  (3) $$DID_{*}=H(pk_{*},ts),\;DDO_{*}=\{DID_{*},\;pk_{*}\}$$
  (4) $$d\in \left[1,\;n-1\right]\;(\mathrm{where}\;n\mathrm{is}\mathrm{the}\mathrm{value}\mathrm{defined}\mathrm{in}\mathrm{secp256k1})$$
  (5) $$\left(x_{1},\;y_{1}\right)=d\cdot P$$
  (6) $$r=x_{1}\;mod\;n$$
  if r≠0:
  (7) $$s=d^{-1}\left(H\left(DDO_{*}\right)+sk_{*}r\right)\;mod\;n$$
  otherwise, repeat from (4)
  (8) $$\sigma_{{*}_{DDO}}=sig_{sk_{*}}\left(DDO_{*}\right)=(r,\;s)$$
- Step 3: The participants create a transaction $DTx_{*}$ using their address $Addr_{*}$, $DDO_{*}$, the VDR’s address $Addr_{VDR}$, $\sigma_{DDO}$, $pk_{*}$ and send $DDO_{*}$ to the VDR.
  (9) $$DTx_{*}=\left\{Addr_{*},\;DDO_{*},\;Addr_{VDR},\;\sigma_{{*}_{DDO}},\;pk_{*}\right\}$$
- Step 4: For verification, the VDR extracts the signature $\sigma_{{*}_{DDO}}$ from $DTx_{*}$ and, if valid, generates a Merkle tree $MT_{*}$ with $DTx_{*}$ and other transactions ${\left\{DTx_{1},\ldots ,DTx_{k}\right\}}_{k\in [1,n]}$ as input. And the VDR records $MT_{*}$.
  The participant registration verification process is as follows.
  If 0<r,s≤n−1:
  (10) $$w=s^{-1}\;mod\;n$$
  (11) $$u_{1}=H\left(DDO_{*}\right)w\;mod\;n$$
  (12) $$u_{2}=rw\;mod\;n$$
  (13) $$\left(x_{1},\;y_{1}\right)=\left(u_{1}\cdot P+u_{2}\cdot pk_{*}\right)\;mod\;n$$
  If $\left(x_{1},\;y_{1}\right)=O$: error message ⊥ (where O is the point of infinity)
  (14) $$v=x_{1}\;mod\;n$$
  (15) $$r\overset{?}{=}v$$
  (16) $$root_{*}=H{\left(...\left(H\left(H\left(H\left(DTx_{*}\right)∥H\left({DTx}_{1}\right)\right)∥...\right)∥H\left({DTx}_{k-1}\right)\right)∥H\left({DTx}_{k}\right)\right)}_{k\in [1,n]}$$
- Step 5: The Holder and Delegatee input their $DID_{*}$, random number $rc_{*}\;\in \;Z_{p}$ to generate a Commitment value $com_{*}$ based on the Pedersen Commitment.
  (17) $$com_{*}=rc_{*}\cdot g_{1}+DID_{*}\cdot g_{2}$$
- Step 6: The Holders and Delegatees send $DID_{*}$, $rc_{*}$, $com_{*}$ to the Issuer, requesting the creation of an anonymous DID, $AID_{*}$.
- Step 7: The Issuer verifies $com_{*}$ and responds with an error message ⊥ if not valid.
  The Holder and Delegatee’s Commitment verification process is as follows:
  (18) $${{com}_{*}}^{\prime }\;=rc_{*}\cdot g_{1}+DID_{*}\cdot g_{2}$$
  (19) $$com_{*}\overset{?}{=}{{com}_{*}}^{\prime }\;$$
  if $com_{*}\neq {{com}_{*}}^{\prime }$: error message ⊥
- Step 8: The Issuer sends $DID_{*}$ to the VDR, requesting the Merkle root value ${root}_{*}$ of the transaction containing the DID. The VDR then sends $root_{*}$ if the corresponding $MT_{*}$ contains $DID_{*}$ as a transaction $DTx_{*}$.
  The Merkle tree registration verification process is as follows:
  If $H(DTx_{*})\in MT_{*}=valid:$ send $root_{*}$.
  Otherwise, error message ⊥.
- Step 9: The Issuer inputs the received ${root}_{*}$, $com_{*}$, pp into NIZK to generate $AID_{*}$.
  (20) $${AID}_{*}=Prove({root}_{*},com_{*},crs)$$
- Step 10: The Issuer transmits the generated $AID_{*}$ to both the Holder and Delegatee.

#### 4.4.3. VC Issuance

The VC Issuance phase involves the Holder sending their anonymous DID and claims to the Issuer and requesting the issuance of a VC. Upon receiving the request, the Issuer verifies the authenticity of the claims received from the Holder and creates a VC if they are valid. The Issuer then issues the generated VC to the Holder.

- Step 1: The Holder sends their $AID_{H}$, $claim_{i},\ldots ,\;claim_{j}$ to the Issuer and requests the issuance of a VC.
- Step 2: The Issuer verifies the authenticity of $claim_{i}$ and, if valid, creates the VC as follows.
  If $claim_{i}=valid$:
  (21) $$h_{claim}=H\left(AID_{H},claim_{i},\ldots ,claim_{j}\right)$$
  (22) $$\sigma_{I_{claim}}=sig_{sk_{I}}\left(h_{claim}\right)$$
  (23) $$VC_{k}={\left\{DID_{I},AID_{H},claim_{i},\ldots ,claim_{j},\sigma_{I_{claim}}\right\}}_{\left(k,\;i,\;j\in [1,n]\right)}$$
  otherwise, error message ⊥.
- Step 3: The Issuer sends the generated $VC_{k}$ to the Holder.
- Step 4: The Holder verifies $\sigma_{I_{claim}}$ in the received $VC_{k}$ using the Issuer’s public key $pk_{I}$. If the verification is correct, the Holder securely stores $VC_{k}$; otherwise, they repeat Step 1 of the VC Issuance.
  If $\sigma_{I_{claim}}=valid$: store $VC_{k}$.
  Otherwise, repeat Step 1 of the VC Issuance.

#### 4.4.4. Delegation

The Delegation phase involves mutual authentication between the Holder and Delegatee, with the Delegatee sending their anonymous DID to the Holder. The Holder then sends their VC and the Delegatee’s anonymous DID to the Issuer, requesting the issuance of a VC for delegation. Subsequently, the Holder combines various VCs and VCs for delegation to create a VP and delegates it to the Delegatee.

- Step 1: The Holder and Delegatee mutually authenticate their $DID_{*}$ via a TLS handshake, and upon successful authentication, the Delegatee securely transmits their $ADID_{D}$ to the Holder.
- Step 2: The Holder sends their $AID_{H}$ and the Delegatee’s $AID_{D}$ to the Issuer, requesting the issuance of a VC for delegation, $DVC_{H\to D}$.
- Step 3: Upon request, the Issuer verifies the authenticity of $AID_{H}$ and $AID_{D}$ and, if valid, creates $DVC_{H\to D}$. The Issuer then issues $DVC_{H\to D}$ to the Holder.
  (24) $$h_{AID}=H\left(AID_{H},AID_{D}\right)$$
  (25) $$\sigma_{I_{AID}}=sig_{sk_{I}}\left(h_{AID}\right)$$
  (26) $$DVC_{H\to D}=\{DID_{I},AID_{H},AID_{D},\sigma_{I_{AID}}\}$$
- Step 4: The Holder, having received the VC for delegation, uses their $VC_{k}$ and $DVC_{H\to D}$ to create $VP_{H\to D}$ and securely delegates it to the Delegatee.
  (27) $$VP_{H\to D}={\left\{VC_{k},DVC_{H\to D}\right\}}_{\left(k\in [1,n]\right)}\;$$

#### 4.4.5. VP Verification

In the VP Verification phase, the Delegatee undergoes authentication verification by the Verifier on behalf of the Holder. For this purpose, the Delegatee creates an RVP (Reconstructed VP) based on sequential aggregate signatures and submits it to the Verifier, who then verifies the RVP and, if valid, provides services to the Delegatee.

- Step 1: The Delegatee requests authentication from the Verifier on behalf of the Holder.
- Step 2: The Verifier requests the necessary credentials to provide the service.
- Step 3: Upon request, the Delegatee combines the appropriate credentials and generates $RVP_{D}$ based on sequential aggregate signatures as follows.
  (28) $$sr\in Z_{p}^{*}$$
  (29) $$A=r^{-1}\cdot P,B=sr\cdot sk_{D}(\prod_{i=1}^{k}VC_{i}DVC_{H\to D}pk_{i})$$
  (30) $$\varsigma_{D}=(A,B)$$
  (31) $$RVP_{D}=\{VP_{H\to D},\varsigma_{D}\}$$
- Step 4: The Delegatee creates an encrypted ciphertext CT using $RVP_{D}$, ${com}_{D}$, ${DID}_{D}$, $rc_{D}$, and the Verifier’s public key, and sends it to the Verifier.
  (32) $$CT=E_{pk_{V}}(RVP_{D},{com}_{D},{DID}_{D},{rc}_{D})$$
- Step 5: The receiving Verifier decrypts the ciphertext CT using their private key.
  (33) $$RVP_{D}=D_{sk_{V}}(CT)$$
- Step 6: The Verifier verifies $com_{D}$ and responds with an error message ⊥ if invalid.
  The Verifier’s commitment verification process is as follows:
  (34) $${{com}_{D}}^{\prime }=rc_{D}\cdot g_{1}+DID_{D}\cdot g_{2}$$
  (35) $$com_{D}\overset{?}{=}{{com}_{D}}^{\prime }$$
  if ${com}_{D}\neq {{com}_{D}}^{\prime }$: error message ⊥.
- Step 7: The Verifier extracts $DID_{I}$ from $RVP_{D}$ and sends $DID_{I}$, $DID_{D}$ to the VDR, requesting $DDO_{I}$, $DDO_{D}$.
- Step 8: The VDR uses DID Resolve to find $DDO_{I}$,$\;DDO_{D}$ mapped to $DID_{I}$, $DID_{D}$ and sends them to the Verifier if a match is found.
- Step 9: The Verifier extracts $pk_{I}$, $pk_{D}$ from $DDO_{I}$, $DDO_{D}$ and verifies $RVP_{D}$ as follows.
  (36) $$C=\prod_{i=1}^{k}VC_{i}DVC_{H\to D}pk_{i}$$
  (37) $$e(A,\;B)\overset{?}{=}e(C,pk_{D})$$

#### 4.4.6. Revocation

In the Revocation phase, if the Holder wishes to revoke a credential previously delegated to the Delegatee, the Holder sends anonymous DIDs and VCs for delegation to the Issuer. The Issuer verifies VCs for delegation and decides on the delegation status. If valid, the Issuer adds this information to the revocation list and updates the list in the VDR.

- Step 1: The Holder sends $AID_{H}$, $AID_{D}$, $DVC_{H\to D}$ to the Issuer, requesting revocation of the delegated credential.
- Step 2: The Issuer verifies if $DVC_{H\to D}$ was issued by them.
- Step 3: If valid, the Issuer adds $AID_{H}$, $ADID_{D}$, $DVC_{H\to D}$, and the revoked date to the revocation list.
  (38) $${RL}^{\prime }=\{AID_{H},AID_{D},DVC_{H\to D},date\}$$
- Step 4: The Issuer sends the updated revocation list to the VDR for recording.
- Step 5: The VDR records ${RL}^{\prime }$.

## 5. Analysis of Proposed Scheme

**Figure 4:**
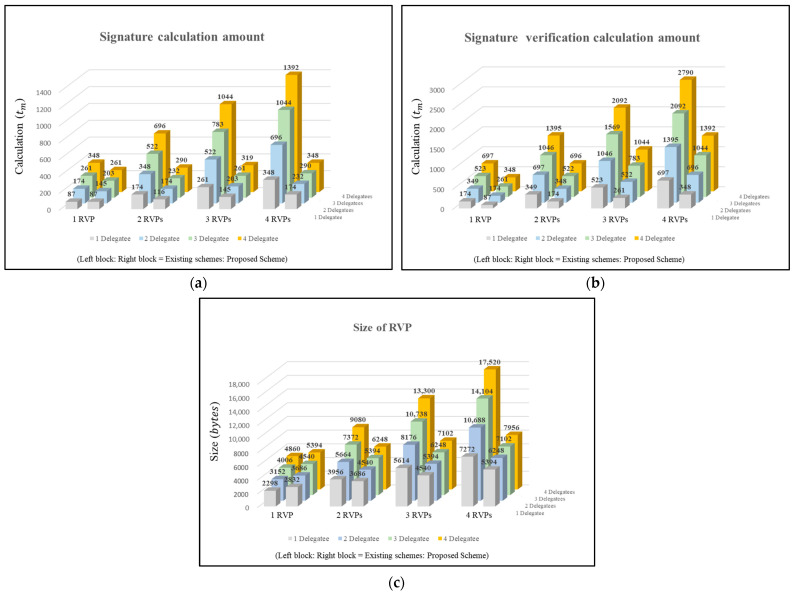
(**a**) Comparison of signature calculation amount; (**b**) comparison of verification calculation amount; (**c**) comparison of RVP sizes.

In this section, the security requirements and additional requirements presented in Section 3 are analyzed, as shown in Table 2, Table 3, Table 4 and Table 5 and Figure 4.

### 5.1. Primary Security Analysis

#### 5.1.1. Delegation

- **Authentication of Delegation**

With DIDs, the Holder must be able to delegate temporarily or permanently some or all of their authority to another entity and confirm that the Holder intended to delegate this authority. To this end, this paper proposed issuing $DVC_{H\to D}$, which includes the Holder’s $AID_{H}$ and Delegatee’s $AID_{D}$, from the Issuer to create $VP_{H\to D}$, as shown in Equations (39)–(42). By delegating the created $VP_{H\to D}$ to the Delegatee, proof of authority delegation can be presented, as shown in Equations (43)–(47).

(39) $$h_{AID}=H\left(AID_{H},AID_{D}\right)$$

(40) $$\sigma_{I_{AID}}=sig_{sk_{I}}\left(h_{AID}\right)$$

(41) $$DVC_{H\to D}=\{DID_{I},AID_{H},AID_{D},\;\sigma_{I_{AID}}\}$$

(42) $$VP_{H\to D}={\left\{VC_{k},DVC_{H\to D}\right\}}_{\left(k\in [1,n]\right)}\;$$

(43) $$A=r^{-1}\cdot P,B=sr\cdot sk_{D}(\prod_{i=1}^{k}VC_{i}DVC_{H\to D}pk_{i})$$

(44) $$\varsigma_{D}=(A,B)$$

(45) $$RVP_{D}=\{VP_{H\to D},\varsigma_{D}\}$$

(46) $$C=\prod_{i=1}^{k}VC_{i}DVC_{H\to D}pk_{i}$$

(47) $$e(A,\;B)\overset{?}{=}e(C,pk_{D})$$

- **Revocation of Delegatee**

The Holder may want to revoke some of the delegated authority to the Delegatee at any time and must be able to cancel such authority delegation. Additionally, if an unauthorized Delegatee makes a request, the Verifier should be able to cryptographically verify the Delegatee’s credentials. Subsequently, the Verifier should provide verification services for illegal authentication requests and be able to block them. To facilitate this, the proposed scheme adds information about $AID_{D}$ and the $VP_{H\to D}$ delegated to the Delegatee to the revocation list ${RL}^{\prime }$ and updates it in the VDR, as shown in Equations (48) and (49).

(48) $${RL}^{\prime }=\{AID_{H},AID_{D},\;DVC_{H\to D},date\}$$

(49) $$RTX_{I}=\left\{Addr_{I},\;{RL}^{\prime },\;Addr_{VDR},\;\sigma_{I_{RL’}},pk_{I}\right\}$$

#### 5.1.2. Anonymity

- **Prevention of Linkage Attack (Unlinkability)**

The proposed scheme is based on the Pedersen Commitment and NIZK, and the Holder or Delegate receives $AID_{H}$ or $AID_{D}$ from the Issuer and includes it in the VC for delegation $DVC_{H\to D}$ to prevent linkage attacks. In other words, each time the Holder provides delegation according to the level of authority to the Delegatee, a VC for delegation containing different $AID_{H}$ and $AID_{D}$ is issued from the Issuer. Afterward, the Holder uses this as input to create a VP and delegates it to the Delegatee. Then, the Delegatee creates an RVP using the received VP and later transmits it to the verifier to perform authentication on its behalf. Through this, even if an attacker obtains various RVPs through illegal routes, the $AID_{H}$ and $AID_{D}$ contained in the RVPs are different, so the attacker can break the connection, which guarantees the anonymity of the Holder and Delegatee.

- **Prevention of Sybil Attack**

The proposed scheme involves the Holder or Delegatee collaborating with the Issuer and the VDR and prevents Sybil attacks based on the Pedersen Commitment, NIZK Proof, and Merkle tree. In the proposed scheme, the Issuer requests the VDR to verify that the actual Holder or Delegatee’s DID is included in the Merkle tree. The requested VDR checks whether the transaction $DTx_{*}$ containing the received Holder or Delegatee’s DID is included in the Merkle tree, as shown in Equations (50) and (51). The VDR then responds to the results $root_{*}$ to the Issuer, and if included, the Issuer generates an $AID_{*}$ with $DID_{*}$, $root_{*}$, and other data as input, as shown in Equations (52)–(55). Afterward, the Holder or Delegatee requests service from the Verifier, and the Verifier performs authentication. However, if the Holder or Delegatee is in doubt, the Verifier may require the Holder to prove their $DTx_{*}$, $AID_{*}$, and the Verifier can cooperate with the VDR to generate $DTx_{*}$, $AID_{*}$ to verify. If the $DTx_{*}$, $AID_{*}$ for this are incorrect, the Verifier may determine that the Holder has a fake identity and refuse service, as shown in Equation (56). Therefore, the proposed scheme can prevent Sybil attacks.

(50) $$H\left({{DTx}_{*}}^{\prime }\right)\in \;MT_{*}\;\overset{?}{=}\;valid$$

(51) $${{root}_{*}}^{\prime }=H{\left(...\left(H\left(H\left(H\left(DTx_{*}\right)∥H\left({DTx}_{1}\right)\right)∥...\right)∥H\left({DTx}_{k-1}\right)\right)∥H\left({DTx}_{k}\right)\right)}_{k\in [1,n]}$$

(52) $${{root}_{*}}^{\prime }\in \;MT_{*}\;\overset{?}{=}\;valid$$

(53) $${{com}_{*}}^{\prime }={{rc}_{*}}^{\prime }\cdot g_{1}+{{DID}_{*}}^{\prime }\cdot g_{2}$$

(54) $$com_{*}\overset{?}{=}{{com}_{*}}^{\prime }$$

(55) $${{AID}_{*}}^{\prime }=Prove({{root}_{*}}^{\prime },\;{{com}_{*}}^{\prime },crs)$$

(56) $${AID}_{*}\overset{?}{=}{{AID}_{*}}^{\prime }$$

### 5.2. Efficiency

For the efficiency analysis in this paper, we compare existing related schemes with the proposed scheme as follows. The paper considers computational complexity as provided in Table 4 and Table 5 [38,39], and analyzes optimization in VC management and minimization in VP storage capacity for the Issuer, Holder, Delegatee, and Verifier.

- **Optimization of VC Management**

This paper assumes a context length of 120 bytes, a DID length of 50 bytes, a claim length of 250 bytes, a signature length of 64 bytes, a proof length of 320 bytes, a VC length of 804 bytes, and a length of 854 bytes for a VC for a delegation intended for delegation.

When creating a single VP based on n VCs for a delegation to provide delegation to m Delegatees, the existing schemes [21,22,32,33] generate with the size being the number of VC for delegation (n) * the number of Delegatee (m) + n∗ m∗ VC for delegation. The existing scheme requires m separate original VCs rather than VCs for delegation. As an exception, in [32], the DID length is 16 bytes.

To optimize VP storage capacity, this paper generates a size of (((n + 1) + m) ∗ VC for delegation) + VC. Additionally, the proposed scheme requires at least two VCs when creating a VP for delegation. However, compared to the existing and reconstructed schemes, the former is small when there is only one VC for delegation, but the latter is efficient when there are many VCs for delegation, or the number of delegates is large.

- **Minimization of VP Storage Capacity**

Existing schemes are identical to creating a VP from a regular DID. In the existing proposed scheme, the Delegatee selects a VC for delegation and generates the Delegatee’s ECDSA-based digital signature as input to create a VP. Existing schemes include at least three signatures (Issuer, Holder, and Delegatee) in the VP, and as the number of VCs included in the VP increases, the digital signature increases by more than two times.

This proposed scheme performs sequential aggregate signatures by inputting the Issuer included in the VP received by the Delegatee from the Holder, the Holder’s digital signature, and the Delegatee’s private key. Afterward, the generated signature and VP are combined to create a Reconstructed VP. Here, by using sequential aggregate signatures, the digital signatures of the Issuer and Holder can be reduced to one. This proposed scheme minimizes the VP storage capacity.

However, when the Holder delegates to a single Delegatee, existing schemes are more efficient than this proposed scheme, but when there are multiple Delegatees, this proposed scheme is more efficient.

- **Minimization of DDO Records in VDR**

This proposed scheme initially creates a DDO when the Holder and Delegatee generate their DID, binding it with the corresponding public key. However, to reduce the burden on the VDR and ensure the anonymity of the Holder or Delegatee, the proposed scheme uses the Pedersen Commitment and NIZK to generate $AID_{H}$, and $AID_{D}$ through the Issuer and includes them in the VP. This allows for verification by the Verifier, minimizing the storage capacity in the VDR.

## 6. Conclusions

This paper presented an innovative approach to reconstructing VPs for a minimally secure delegation of authority using schemes such as SAS, NIZK Proof, and Merkle tree to maintain anonymity and prevent Sybil attacks in DIDs. These measures ensure that users’ identities remain protected even when multiple VPs are analyzed, addressing the crucial challenge of preserving self-sovereignty in the digital era.

Additionally, the proposed scheme proposed optimizations to manage VCs and minimize VP storage requirements to address the efficiency and security issues of the delegation process. This strategy mitigates security risks related to delegation and anonymity, efficiently reduces the computational and verification efforts for signatures, and reduces the size of the VP by about 1.2 to 2 times. In other words, this enhances privacy protection and respects individuals’ autonomy over their digital identities, reinforcing the right to self-determination.

Future works will refine and evaluate the proposed scheme’s performance to meet evolving security needs and offer a robust and reliable framework for digital identity management in the DID ecosystem.

## Figures and Tables

**Table 1:** System parameters.

Symbol	Definition
*	Participant entities mean the Holder, Issuer, and Verifier collectively
H, I, V, D, VDR	Holder, Issuer, Verifier, Delegatee, and Verifiable Data Registry
p, q	Large prime numbers
P	Elliptic curve base point
H()	Hash function, $H:\;{\left\{0,1\right\}}^{*}\to Z_{p}^{*}$
$G_{1},\;G_{2}$	Addition group and multiple group
$G_{T}$	Groups in bilinear mapping
$g_{1},\;g_{2}$	Generator of $G_{1},\;G_{2}$
e	Bilinear map, $e∶G_{1}\times G_{2}⟶G_{T}$
crs	Common reference string
PP	Public parameters
$sk_{*},\;pk_{*}$	Participant’s private key and public key pair
ts	Timestamp
$DID_{*},\;DDO_{*}$	Participant’s DID and DID document
$DTx_{*}$	Transaction that contains participant’s DDO
$Addr_{*}$	Participant’s blockchain network address
$\sigma_{{*}_{input}}$	Signed value of the participant’s input message
$MT_{*}$	Binary tree structure with hash values generated including $DTx_{*}$ as the upper nodes.
$root_{*}$	Top Merkle root value of a Merkle tree
Prove()	Function that generates a proof value based on NIZK Proof
$AID_{*}$	Participant’s anonymous DID
$claim_{H}$	Holder’s claim (s)
$VC_{H\to D}$	Holder’s verifiable credential for Delegatee
$VP_{H}$	Holder’s verifiable presentation
$DVC_{H\to D}$	Holder’s verifiable credential for Delegatee
ς	Signature using sequential aggregate signature
$RVP_{D}$	Delegatee’s re-structure verifiable presentation
CT	ECC (Elliptic-Curve Cryptography)-based public key ciphertext for message m for secure communication
date	Current date and time
RL	Revocation list

**Table 2:** Analysis table of proposed scheme for delegation.

Requirements	Sabadello et al. [25]	Boneh et al. [26]	Lysyanskaya et al. [27]	Proposed Scheme
Authentication of Delegation	Delegatee’s DID in VC	Delegatee’s DID in VC	Delegatee’s DID in DDO	Delegatee’s DID in VC and SAS
Prevention of Impersonation Attack	PKI	PKI	PKI	PKI, Commitment, NIZK
Revocation of Delegatee	X	Δ (Timestamp)	Δ (Timestamp)	Revocation List
Prevention of Linkage Attack	X	X	X	NIZK, Merkle Tree

Δ: Some provided, X: not provided.

**Table 3:** Analysis table of proposed scheme for anonymity.

Requirements	Song [21]	Yin et al. [22]	Kim [32]	Bosk et al. [33]	Proposed Scheme
Unforgeability	DSA	DSA and sign the digest of the claims	DSA	PS Signature	ECDSA, SAS
Unlinkability (DID)	Commitment and NIZK Proof	X	sDID	NIZK, Aggregate, and PS Signature	Commitment and NIZK Proof
Privacy	Commitment, NIZK, and Merkle tree	Δ (Commitment, AND, OR, and Range Proof)	Δ	NIZK, Aggregate, PS Signature	Commitment, NIZK, and Merkle tree
Prevention of Sybil Attack	Commitment, NIZK Proof, Merkle tree	Registration through Issuer	X	X	Commitment, NIZK Proof, and Merkle tree
Minimization of DDO Records in VDR	Low	-	High	Low	Low

Δ: Some provided, X: not provided.

**Table 4:** Analysis table of proposed scheme for efficiency comparison.

Requirements	Sabadello et al. [25]	Boneh et al. [26]	Lysyanskaya et al. [27]	Proposed Scheme
Sign	3nmEM	3nmEM	3nmEM	(n+2m)EM
Verify	3nm(EA+2EM)	3nm(EA+2EM)	3nm(EA+2EM)	nmP

*n*: Number of VCs for delegation, *m*: number of Delegatees, *EA*: elliptic curve addition, *EM*: elliptic curve multiplication, *P*: pairing.

**Table 5:** Computational amount parameters.

Symbol	Definition	Calculation Amount
$t_{m}$	Time of modular multiplication	$t_{m}$
E	Exponentiation	$\approx 240\;t_{m}$
EM	Elliptic curve point multiplication	$\approx 29\;t_{m}$
P	Bilinear pairing	$\approx 87\;t_{m}$

## Data Availability

Data is contained within the article.
